# Atypical Presentation of Lip Nodules in Clinically Diagnosed Juvenile Hyaline Fibromatosis

**DOI:** 10.7759/cureus.40512

**Published:** 2023-06-16

**Authors:** Ee Theng Khoo, Ju Ann Tan, Muath Mamdouh Mahmod Al-Chalabi, Mohammad Ali Mat Zain, Wan Azman Wan Sulaiman

**Affiliations:** 1 Reconstructive Sciences Unit, Universiti Sains Malaysia (USM), Kota Bharu, MYS; 2 Department of Plastic and Reconstructive Surgery, Hospital Kuala Lumpur, Kuala Lumpur, MYS; 3 Reconstructive Sciences Unit, Kota Bharu, MYS

**Keywords:** juvenile hyaline fibromatosis, lip nodules, extensive lip nodules, perioral nodules, autosomal recessive disease

## Abstract

Juvenile hyaline fibromatosis (JHF) is a rare, hereditary disease characterized by abnormal hyaline deposits within the skin, soft tissues, joints, and bones. The condition itself is often debilitating, with no curative treatment available. A definitive diagnosis is established by genetic testing. However, the hallmarks of gingival hypertrophy, subcutaneous scalp nodules, and joint contractures can be used as a clinical guide when genetic testing is unavailable. Here, we report an unusual case of a five-year-old child clinically diagnosed with juvenile hyaline fibromatosis with atypical nodules exclusively confined to the perioral region.

## Introduction

Juvenile hyaline fibromatosis (JHF) is a rare, autosomal recessive disorder linked to a capillary morphogenesis protein 2 (CMG2) gene mutation located on chromosome 4q21. This mutation sequentially promotes the abnormal deposition of hyalinized tissue [1].

In 1873, John Murray first described JHF in a report, "Three peculiar cases of Molluscum fibrosum in children" [2]. Only 84 cases of JHF have been reported worldwide, with no gender predilection [3]. Affected individuals were also reported to be born from consanguineous marriages [4-6]. The first clinical manifestation can be seen from two months to four years old with normal neurocognitive development. Subsequent manifestations tend to intensify with age, with no curative treatment available. Their lifespan has been estimated to be at least four decades [7-9].

Commonly reported clinical features of JHF include subcutaneous skin nodules (85.7%), gingival hypertrophy (92.9%), and joint contractures (95.2%). Cutaneous findings of pearly plaques have also been reported over the perianal region (48.8%), as well as the forehead, nasolabial folds, ears, and posterior neck (42.9%) [2,3,7]. Based on the clinical presentation, a grading system has been introduced to delineate the severity of the disease and its prognosis. There are four grades of JHF, which include mild (skin and/or gingival involvement), moderate (joints and/or bone involvement), severe (internal organ involvement with or without clinical manifestation), and lethal (organ failure and/or septicemia) [10].

Herein, we report a case of clinically diagnosed JHF with an atypical finding of extensive involvement of lip nodules, which requires surgical intervention to alleviate the oral obstruction. So far, no JHF cases report similar findings of multiple extensive lip nodule involvement.

## Case presentation

**Figure 1:**
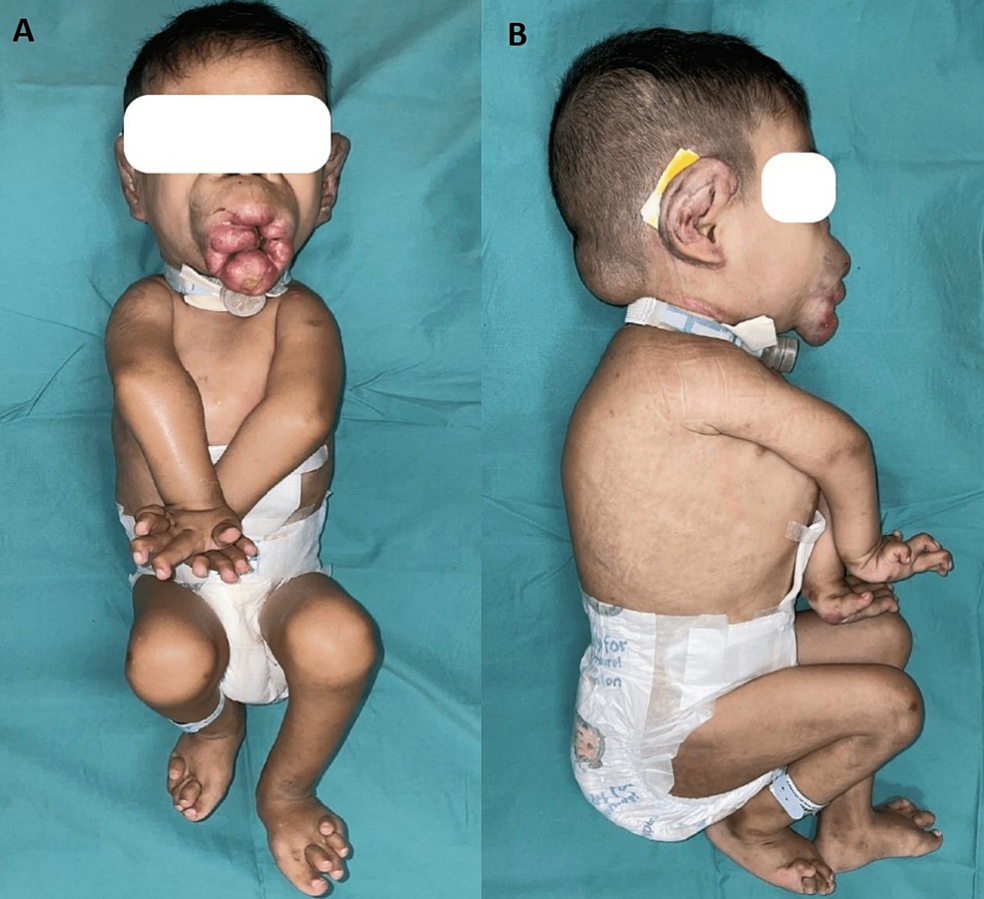
Front (A) and side (B) views show large nodules over the lips, ear lobules involvement, scalp nodule, and severe flexion joint contractures.

A five-year-old Malay boy presented with large, painless nodules over the lips, ear lobules, and scalp, gingival hypertrophy, and severe flexion joint contractures (Figure 1).

He is the youngest child of three siblings and was born to a second-degree consanguineous marriage. Both his parents and two siblings were unaffected. The antenatal history was unremarkable, and he was delivered at term with no notable congenital disabilities. He presented with gingival hypertrophy at three months and reduced joint movements over the upper extremities. Joint stiffness continued to manifest, involving both upper and lower extremities by the age of six months. Multiple small lip nodules were seen at the age of one. Otherwise, he has no history of persistent diarrhea or recurrent chest infections. During a medical consultation, juvenile hyaline fibromatosis was diagnosed clinically because the family could not afford the cost of genetic testing.

**Figure 2:**
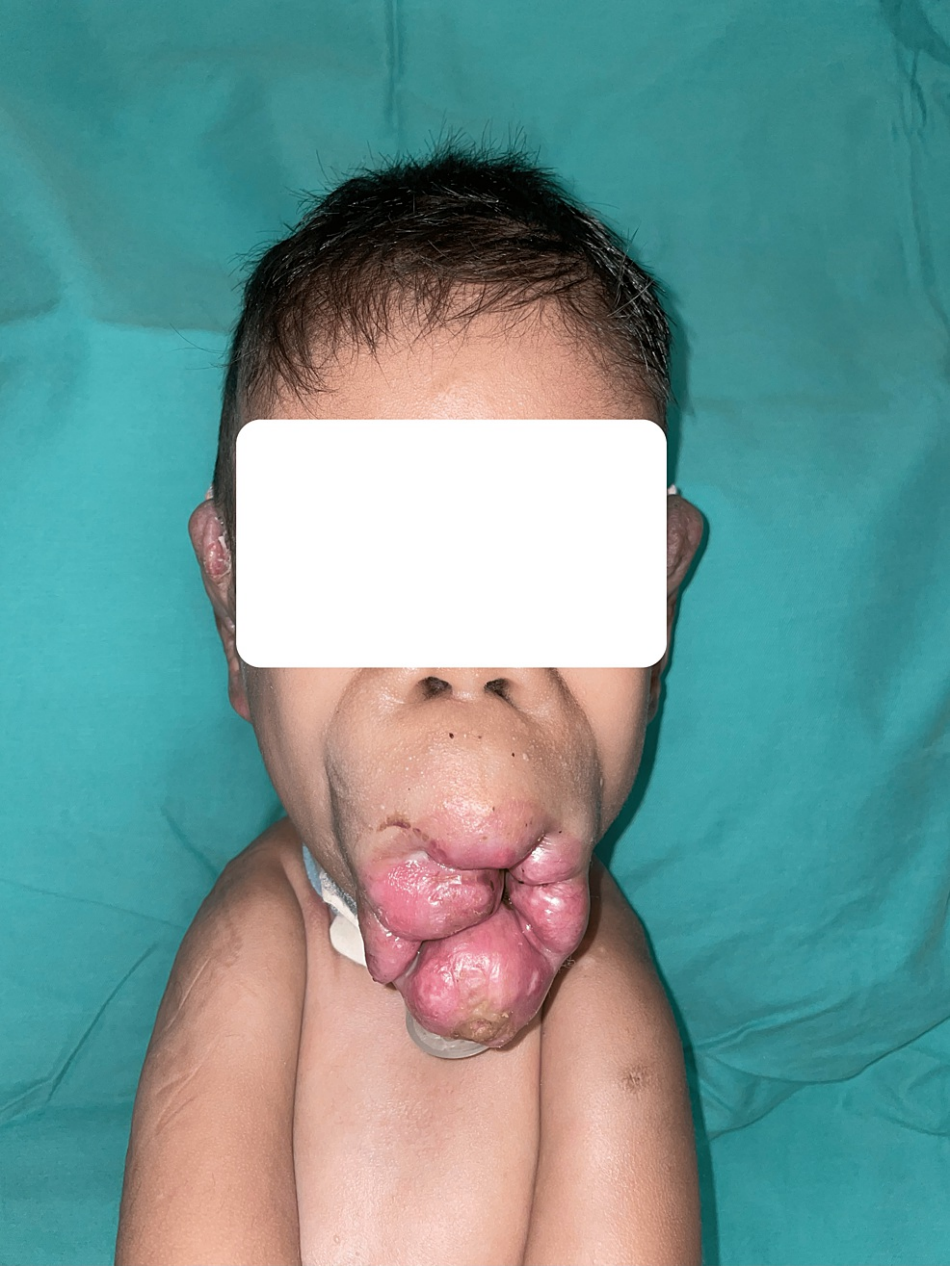
Extensive perioral lip nodules.

Over three years, the nodules over the lips increased in size (Figure 2) with extensive gingival hypertrophy. Soon after, the patient became bedridden due to progressive contractures over the upper and lower limbs.

He underwent prophylactic tracheostomy, percutaneous endoscopic gastrostomy (PEG) insertion, and gingivectomy at the age of three. He could tolerate food orally with supplementary nourishing fluid via a PEG tube. Unfortunately, the slow-growing lip nodules eventually led to complete obstruction of the oral cavity. He was then referred to the Plastic and Reconstructive Surgery team for debulking of the lip nodules.

**Figure 3:**
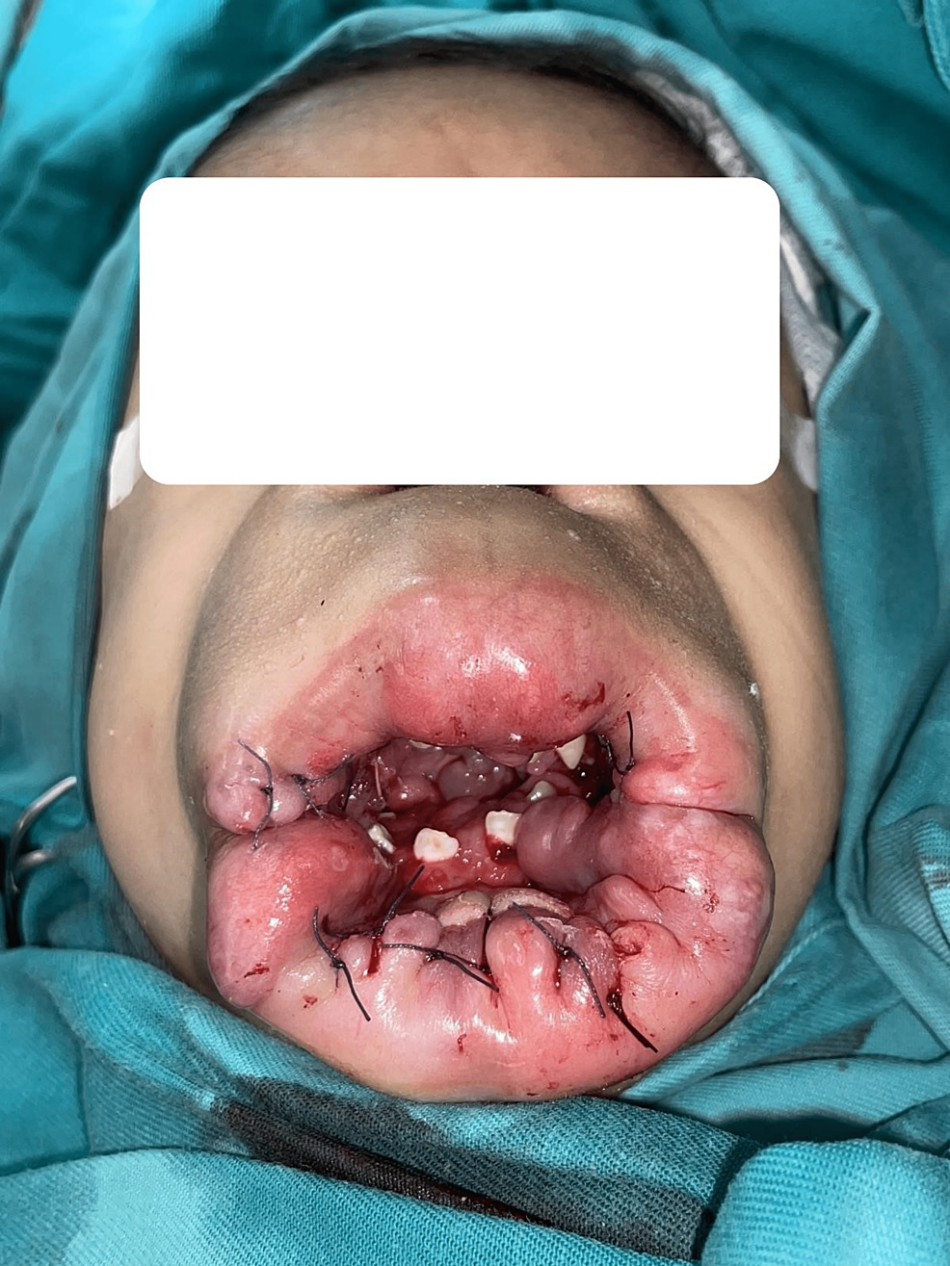
Post-debulking of the lip nodules.

During the clinic review, the examination showed multiple large nodules over the upper and lower lips with gingival hypertrophy. The surrounding nasal and oral tissues were thickened. Pearly papules were appreciated over the nasal region. Both external ears were hypertrophic and deformed. A 5-cm single scalp nodule was seen over the occipital region. Both the trunk and extremities were spared from skin thickening or cutaneous nodules. Severe flexural contractures can be seen on both upper and lower limbs, involving the shoulders, elbows, wrists, fingers, hips, knees, ankles, and toes. Other systemic examinations were unremarkable. Routine blood investigations were within normal limits. Subsequently, he underwent debulking of the lip nodules to alleviate the obstruction to the oral cavity (Figure 3).

## Discussion

Affected individuals with JHF may present with various clinical manifestations, including cutaneous, mucosal, osteoarticular, muscular, internal organ involvement, and systemic decompensation. A definitive diagnosis of JHF can be established based on the identification of a CMG2 gene mutation during molecular genetic testing [1]. However, it is not readily accessible, and the cost can be burdensome to many.

Most confirmed JHF patients reported typical hallmarks of severe gingival hypertrophy, progressive joint contractures, and localized subcutaneous nodules favoring the scalp, trunk, and extremities [3,11]. Our patient was diagnosed clinically based on these strong resemblances and features of typical JHF, as mentioned above.

Interestingly, he also presents with an atypical location of nodules, exclusively in the perioral region. A feature that has not been reported elsewhere. As the disease progressed with irreversible flexural joint contractures, our patient became utterly dependent, from feeding to self-care. The growing perioral nodules became a concerning issue for both parents, impeding the child’s oral feeding.

Early surgical excision of small, resectable nodules is preferred to address functional or aesthetic problems [4,5]. However, recurrences have been reported [3,9]. Our patient was offered debulking surgery as part of symptomatic management to alleviate the oral obstruction. Attempts to excise the nodules completely would be as crippling as the disease. There is also the possibility of future overgrowth of new lip nodules, which may require repeated surgical intervention. Hence, long-term follow-up is required to assess the outcome.

## Conclusions

Extensive perioral nodules are a relatively uncommon involvement in JHF, as they have not been reported in the literature. This finding can provide an additional expansion to the commonly reported features of JHF. Due to the rarity of the disease itself, no definitive treatment has proven successful. Hence, treatment modalities should be tailored on a case-by-case basis. Early surgical excision and rehabilitation measures should be enforced as soon as possible to reduce the degree of functional impairment.
